# Liquid chromatography coupled to mass spectrometry metabolomic analysis of cerebrospinal fluid revealed the metabolic characteristics of moyamoya disease

**DOI:** 10.3389/fneur.2024.1298385

**Published:** 2024-02-15

**Authors:** Jin Yu, Tongyu Chen, Xiang Li, Jincao Chen, Wei Wei, Jianjian Zhang

**Affiliations:** ^1^Department of Neurosurgery, Zhongnan Hospital of Wuhan University, Wuhan, Hubei, China; ^2^Brain Research Center, Zhongnan Hospital of Wuhan University, Wuhan, Hubei, China

**Keywords:** moyamoya disease, metabolomics, cerebrospinal fluid, purine metabolism, pyrimidine metabolism

## Abstract

**Objective:**

Metabolomics has found extensive applications in the field of neurological diseases, significantly contributing to their diagnosis and treatment. However, there has been limited research applying metabolomics to moyamoya disease (MMD). This study aims to investigate and identify differential metabolites associated with MMD.

**Methods:**

We employed a liquid chromatography coupled with mass spectrometry (LC-MS) approach, complemented by univariate and multivariate analyses, to discern metabolic biomarkers in cerebrospinal fluid samples. We then compared these biomarkers between MMD patients and healthy controls (Ctl).

**Results:**

Sixteen patients diagnosed with MMD via cerebral angiography and eight healthy controls were enrolled in this study. Comparative analyses, including univariate and multivariate analyses, correlation studies, heatmaps, Volcano Plots, and KEGG pathway enrichment, were performed between MMD patients and controls. As a result, we identified 129 significant differential metabolites in the cerebrospinal fluid between MMD patients and controls. These metabolic biomarkers are associated with various pathways, with notable involvement in purine and pyrimidine metabolism.

**Conclusion:**

Utilizing an LC-MS-based metabolomics approach holds promise for enhancing the clinical diagnosis of MMD. The identified biomarkers offer potential avenues for the development of novel diagnostic methods for MMD and offer fresh insights into the pathogenesis of the disease.

## Introduction

Moyamoya disease (MMD) is defined by the gradual narrowing or blockage at the terminal segments of the bilateral internal carotid artery (ICA) or the initial portions of the anterior and middle cerebral arteries. This condition is often accompanied by compensatory expansion of the perforating arteries and the development of intricate vascular networks known as “moyamoya vessels” (1). MMD has been documented worldwide, with a notably higher prevalence in East Asian nations, whereas it is infrequently reported in individuals of Caucasian descent (2). Although the epidemiology, clinical features, and treatment of MMD have been extensively studied, the etiology and pathogenesis remain largely unknown. It is believed that MMD is caused by an intricate mechanism involved in inflammatory, angiogenic, and vasculogenic pathway abnormalities in genetically susceptible individuals (3). Lack of a comprehensive understanding of the causes and mechanisms behind MMD has hindered the development of effective early prevention and intervention strategies for the condition. At present, the diagnosis of MMD mainly relies on digital subtraction angiography (DSA) (4), but DSA is an invasive operation and relatively high-cost, which is not conducive to its application in large-scale routine examination of MMD. Therefore, the search for potential biological indicators will be beneficial to explore the pathogenesis of MMD and develop new clinical tests.

Metabolomics is an evolving discipline within the life sciences that employs advanced analytical chemistry methods in conjunction with intricate statistical approaches to comprehensively examine the distinctive characteristics of disease initiation and progression (5). Metabolomics has been widely used in several neurological diseases such as glioblastoma, Parkinson's disease, cerebral Ischemia, intracranial aneurysm, etc. (6–9), which has provided great help for the diagnosis and treatment of these diseases. However, there were only three metabolomic studies on MMD patients conducted up to now. Among them, two studies (10, 11) were focused on the serum metabolites in MMD and only one (12) on cerebrospinal fluid (CSF). As moyamoya vessels are exclusive to the cerebrovascular system and are not observed in the external carotid system or other organs, the brain's microenvironment may have a significant influence on MMD. Analyzing the specific metabolomics of MMD using CSF as a specimen is a plausible approach.

In this study, our objective was to detect distinctive metabolites linked to MMD. We achieved this by employing a methodology that combines liquid chromatography coupled with mass spectrometry (LC-MS) with both univariate and multivariate analyses. These results could be beneficial to explore new diagnostic methods of MMD and provide a new understanding of the pathogenesis of MMD.

## Methods and materials

### Participants

We recruited a total of 36 patients who had been diagnosed with MMD through cerebral angiography between January 2021 and June 2022 at Zhongnan Hospital of Wuhan University. All patients satisfied the diagnostic criteria of the Research Committee on Spontaneous Occlusion of the Circle of Willis of the Ministry of Health, Labor, and Welfare, Japan (13–15). Our study excluded MMD patients with comorbid conditions such as hypertension, diabetes, and coronary artery disease. Healthy controls came from colorectal patients who were excluded from any cerebral disease and other tumor diseases. As a result, our final study cohort consisted of 16 MMD patients and 8 healthy controls. To minimize errors attributed to basal metabolism, the individuals with MMD participating in our study were those admitted to the hospital, having maintained a light diet for 3 days preceding the operation. Likewise, the dietary habits of healthy individuals were documented for 3 days preceding their enrollment to mitigate potential errors associated with dietary factors (10). CSF samples (5 ml) were collected from all MMD patients during their bypass operation, as well as from all controls during their lumbar anesthesia. These samples were subsequently subjected to centrifugation at 5,000 rpm for 10 min at 4°C and then stored at −80°C until further analysis.

### Metabolite extraction process

To extract metabolites, 100 μL of the samples were placed into Eppendorf tubes and reconstituted with prechilled 80% methanol using thorough vortexing. The samples were then incubated on ice for 5 min and subsequently centrifuged at 15,000 *g* and 4°C for 20 min. A portion of the resulting supernatant was diluted to a final concentration containing 53% methanol using LC-MS grade water. These samples were then transferred to fresh Eppendorf tubes and underwent another centrifugation at 15,000 *g* and 4°C for 20 min. Finally, the supernatant was injected into the LC-MS/MS system for analysis.

### UHPLC-MS/MS analysis

For UHPLC-MS/MS analysis, we utilized a Vanquish UHPLC system (Thermo Fisher, Germany) coupled with an Orbitrap Q ExactiveTM HF mass spectrometer (Thermo Fisher, Germany) at Novogene Co., Ltd. (Beijing, China). The samples were injected into a Hypesil Gold column (100 × 2.1 mm, 1.9 μm) and subjected to a 12-min linear gradient at a flow rate of 0.2 mL/min. In the positive polarity mode, eluent A (0.1% FA in water) and eluent B (methanol) were used, while in the negative polarity mode, eluent A consisted of 5 mM ammonium acetate at pH 9.0, and eluent B was methanol. The solvent gradient was set as follows: 2% B for 1.5 min, a gradient from 2 to 85% B over 3 min, 85 to 100% B over 10 min, holding at 100% B for 10.1 min and finally returning to 2% B over 12 min. The Q ExactiveTM HF mass spectrometer was operated in positive/negative polarity mode with a spray voltage of 3.5 kV, a capillary temperature of 320°C, a sheath gas flow rate of 35 psi, an auxiliary gas flow rate of 10 L/min, an S-lens RF level of 60, and an auxiliary gas heater temperature of 350°C.

### Data processing and metabolite identification

The raw data files generated by UHPLC-MS/MS were subjected to data processing using Compound Discoverer 3.1 (CD3.1, Thermo Fisher). This processing involved peak alignment, peak picking, and quantitation for each metabolite. The key parameters were configured as follows: retention time tolerance of 0.2 min, actual mass tolerance of 5 ppm, signal intensity tolerance of 30%, signal/noise ratio set to 3, and a minimum intensity threshold, among others. Subsequently, peak intensities were normalized relative to the total spectral intensity. The normalized data were employed for predicting molecular formulas based on additive ions, molecular ion peaks, and fragment ions. Subsequently, peaks were cross-referenced with databases such as mzCloud (https://www.mzcloud.org/), mzVault, and MassList to obtain precise qualitative and relative quantitative results. Statistical analyses were conducted using the R statistical software (R version R-3.4.3), Python (Python 2.7.6 version), and CentOS (CentOS release 6.6). In cases where data did not exhibit a normal distribution, attempts were made to normalize the data using the area normalization method.

### Data analysis

The identification of these metabolites was accomplished by referencing the KEGG database (https://www.genome.jp/kegg/pathway.html), HMDB database (https://hmdb.ca/metabolites), and LIPIDMaps database (http://www.lipidmaps.org/). Subsequently, we conducted Principal Components Analysis (PCA) and Partial Least Squares Discriminant Analysis (PLS-DA) using metaX14 (16), a versatile and comprehensive software for processing metabolomics data. We employed univariate analysis (*t*-test) to determine statistical significance (*P*-value). Metabolites meeting the criteria of Variable Importance in Projection (VIP) > 1, *P*-value < 0.05, and fold change ≥2 or FC ≤ 0.5 were considered differential metabolites. To visualize these metabolites of interest, Volcano plots were generated based on the log2 (Fold Change) and -log10 (*p*-value) using the ggplot2 package in the R programming language. For clustering heat maps, data normalization involved *z*-scores of the intensity areas of differential metabolites, and the heat maps were generated using the Pheatmap package in the R language. The correlation among differential metabolites was assessed using the “cor()” function in R (method=pearson), and statistically significant correlations between differential metabolites were determined through “cor.mtest()” in R. A *P*-value < 0.05 was considered statistically significant, and correlation plots were constructed using the corrplot package in R. To gain insights into the functions of these metabolites and their involvement in metabolic pathways, we referenced the KEGG database. Furthermore, we conducted metabolic pathway enrichment analysis, considering pathways as enriched when the ratio met the condition x/n > y/N, and pathways were considered statistically significantly enriched when the *P*-value for the pathway was < 0.05.

## Results

### Basic clinical characteristics

In this study, a total of 16 patients diagnosed with MMD and 8 healthy controls (HCs) were included. We summarized the fundamental clinical characteristics in Table 1. There were no significant differences between MMD patients and HCs in terms of age and gender (all *P* > 0.05). Among the MMD patients, the onset types were divided into ischemic (eight cases) and hemorrhagic (eight cases). Additionally, there were 6 cases of bilateral MMD and 10 cases of unilateral MMD.

**Table 1:** Clinical characteristics of enrolled moyamoya disease patients (MMD) and healthy control (Ctl).

	**Ctl (*n* = 8)**	**MMD (*n* = 16)**	***P*-value**
Age (yrs)	52.25 ± 8.42	54.19 ± 6.97	0.763
Female	4	5	0.694
Onset-Ischemia	-	8	N/A
Onset-Hemorrhage	-	8	N/A
Type-Bilateral	-	6	N/A
Type-Unilateral	-	10	N/A

### Metabolite analysis

**Figure 1:**
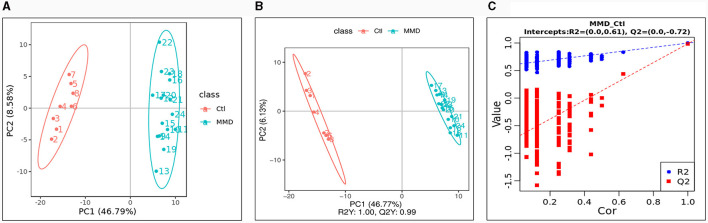
Multivariate statistical analysis between the control group (Ctl) and moyamoya disease group (MMD). **(A)** Principal components analysis (PCA) scores plot; **(B)** partial least squares discriminant analysis (PLS-DA) scores plot; **(C)** Permutations Plot.

We employed the LC-MS method to analyze all CSF samples obtained from both the MMD and control groups. After conducting an analysis of unknown compounds and performing quantitative assessments, the identified metabolites within each CSF sample were used for subsequent multivariate analysis. The Principal Component Analysis (PCA) scores plot revealed a distinct difference between the MMD and healthy control groups (Figure 1A). Furthermore, Partial Least Squares Discriminant Analysis (PLS-DA) reaffirmed this discrepancy, yielding an R2Y score of 1.00 and a Q2Y score of 0.99 (Figure 1B). To ensure the validity of the constructed PLS-DA model and guard against overfitting, we conducted a permutation test. The original R2 and Q2 values on the right side of the plot were significantly higher than their corresponding permuted values on the left, with R2 = 0.61 and Q2 = −0.72 (Figure 1C). This multivariate statistical analysis indicated that the grouping was justifiable, and the model maintained its credibility.

### Differential metabolite analysis

**Figure 2:**
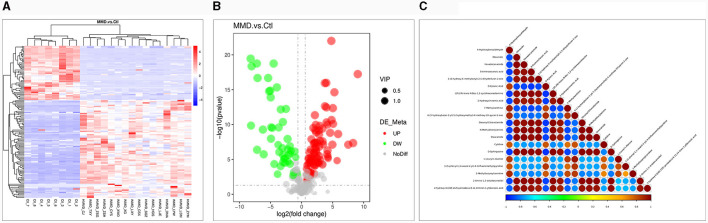
Differential metabolite analysis. **(A)** Heat map for identified metabolites in moyamoya disease (MMD) patients and healthy controls (Ctl). The color of each section is proportional to the significance of change of metabolites (red, upregulated; blue, downregulated). Rows, samples; columns, metabolites; **(B)** Volcano Plot to distinguish metabolites with significant difference (VIP > 1.0, *P* < 0.05) between the two groups. The color of each dot is proportional to the significance of change of metabolites (red, upregulated; blue, downregulated; gray, no difference); **(C)** Correlation analysis of the differential metabolites MMD patients and Ctl. *P*-value < 0.05 was used as the threshold of significant correlation. The highest correlation is 1, which is a complete positive correlation (red); the lowest correlation is −1, which is a complete negative correlation (blue). The figure shows the correlation of the top 20 differential metabolites ranked from smallest to largest by *P*-value.

The metabolic data were analyzed and presented in a clustering heatmap. As depicted in Figure 2A, the majority of samples distinctly grouped into two separate clusters, underscoring the presence of markedly distinct metabolic characteristics in MMD patients compared to healthy individuals. The Volcano Plot in Figure 2B displayed 129 metabolites exhibiting significant differences (VIP > 1.0, *P* < 0.05) between the two groups. Further details about these metabolites with significant differences can be found in Supplementary material 1. Typically, different metabolites exhibit synergistic or mutually exclusive relationships. To assess the consistency in the trend of changes among metabolites, we conducted an analysis of correlations between all metabolites, calculating the Pearson correlation coefficient for each pair. Figure 2C illustrates clear correlations among the majority of the differential metabolites.

### Analyses of metabolic pathways

**Figure 3:**
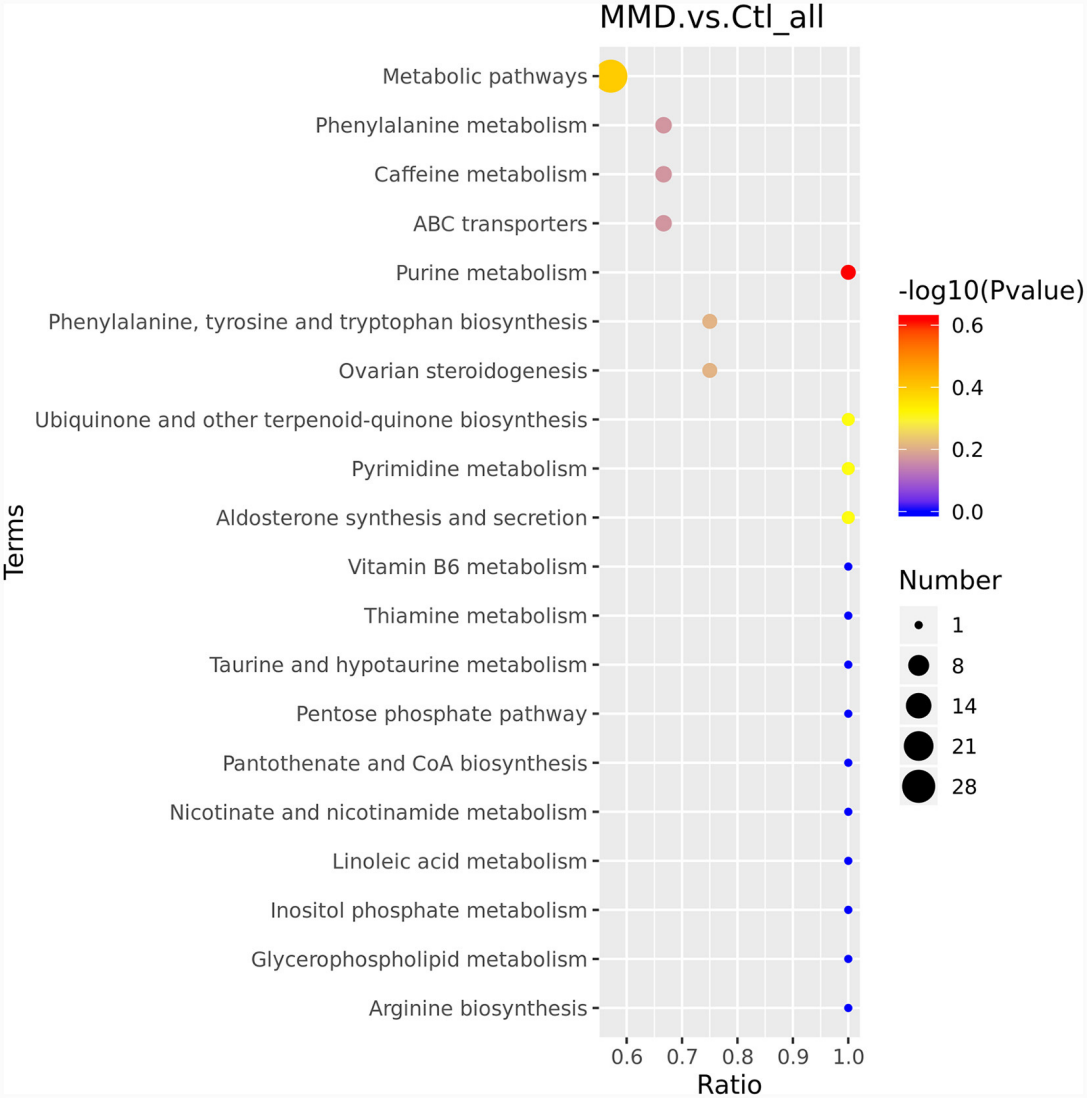
KEGG enriched bubble map. The abscissa is x/y (the number of differentiated metabolites in the corresponding metabolic pathway/the total number of identified metabolites in the pathway). The color of the points represents the *P*-value of the hypergeometric test. The size of the dots represents the number of differentiated metabolites in the corresponding pathway.

To delve deeper into the functions of the identified metabolites and their association with metabolic pathways, we turned to the KEGG database to perform metabolic pathway enrichment analysis on the differential metabolites. This analysis revealed several pathways that are closely related to the progression of MMD, as depicted in Figure 3. The top 10 pathways, which potentially involve affected metabolites, are outlined in Table 2 and Supplementary material 2.

**Table 2:** Top 10 results from pathway analysis.

**Pathway**	***P*-value**	***x***	***y***	***n***	***N***
Purine metabolism	0.241615	3	3	37	70
Metabolic pathways	0.40316	28	49	37	70
Ubiquinone and other terpenoid-quinone biosynthesis	0.49441	2	2	37	70
Pyrimidine metabolism	0.49441	2	2	37	70
Aldosterone synthesis and secretion	0.49441	2	2	37	70
Phenylalanine, tyrosine, and tryptophan biosynthesis	0.61648	3	4	37	70
Ovarian steroidogenesis	0.61648	3	4	37	70
Caffeine metabolism	0.676675	4	6	37	70
Phenylalanine metabolism	0.676675	4	6	37	70
ABC transporters	0.676675	4	6	37	70

x, the number of differential metabolites associated with this pathway; y, The number of background (all) metabolites associated with this pathway; n, The number of differential metabolites annotated by KEGG; N, The number of background (all) metabolites for KEGG's annotation.

## Discussion

MMD is radiologically characterized by progressive stenosis/occlusion of major cerebral arteries caused by intimal hyperplasia and the formation of compensatory capillary collaterals due to a compensation for cerebral ischemia and hypoxia (17, 18). Numerous biological processes are involved, such as dysfunction of endothelial lineage cells, smooth muscle cells and altered levels of various cytokines chemokines and growth factors in patient-derived samples (like VEGF, FGF, platelet-derived growth factor, and hepatocyte growth factor and inflammation-related cytokines) (18). All these processes require the involvement of complex metabolites, suggesting that patients with MMD should have special metabolomic characteristics. Indeed, the first metabolomic study was conducted in 2015 by Jeon et al. (12), by utilizing hydrogen nuclear magnetic resonance spectroscopy on CSF, it was observed that bilateral MMD) exhibited elevated levels of glutamine and taurine while showing decreased levels of glucose, citrate, and myo-inositol compared to individuals with atherosclerotic cerebrovascular disease (ACVD). On the other hand, unilateral MMD exhibited higher concentrations of glutamine and taurine and lower levels of glutamate in comparison to ACVD. This study initiated the study of metabolomics in MMD, but due to the current detection technology, more MMD-related metabolites need to be further studied. Five years later, Geng et al. conducted a study in which they employed a non-targeted gas chromatography–mass spectrometry (GC–MS) approach. In their research, they successfully identified 25 distinctive serum metabolic biomarkers that differentiated between MMD patients and healthy controls. These metabolic biomarkers play roles in various pathways and are strongly linked to the metabolism of amino acids, lipids, carbohydrates, and carbohydrate translation (10). In their 2022 study, the authors also presented a comprehensive overview of amino acid alterations in the serum of MMD patients. Notably, they identified significant variations in the levels of 12 amino acids between the MMD patients and healthy controls (HCs). Furthermore, through ROC curve analysis, the authors pinpointed four amino acid biomarkers (L-methionine, L-glutamic acid, β-alanine, and o-phosphoserine) that demonstrated exceptional sensitivity and specificity in distinguishing MMD patients from HCs. All these studies have made great efforts to reveal the metabolic characteristics of MMD. However, our understanding of the metabolic characteristics of MMD remains inadequate compared with the increasing number of MMD patients.

As a chronic cerebrovascular disease, MMD can lead to the change of intracranial perfusion and cause cerebral ischemia which triggers sequential and complex metabolic and cellular pathologies (19). CSF could be a reasonable specimen to reflect these intracranial changes. However, no studies have been conducted using CSF samples for metabolomic analysis in mmd patients and healthy controls. In this study, we explored differential metabolites from CSF between MMD patients and healthy controls. The differential metabolite analysis found a clear difference between MMD patients and healthy controls. A total of 129 metabolites with noticeable differences were identified and enriched in several pathways.

Among the enriched pathways, purine metabolism and pyrimidine metabolism aroused our interest. Purines and pyrimidines are among the most abundant metabolic substrates in biological systems. Apart from their fundamental role as the building blocks for DNA and RNA, purines and pyrimidines, as well as purinergic signaling molecules like adenosine (P1) and ATP and ADP (P2), and their corresponding receptors, play essential roles in various cellular processes. These processes encompass cell proliferation, differentiation, and programmed cell death, all of which are pivotal in the context of development and tissue regeneration (20). MMD involves intimal hyperplasia and abnormal angiogenesis and requires increased synthesis of purines and pyrimidines to obtain an adequate nucleotide pool to meet the demands of DNA replication and RNA production during the progression of proliferative lesions. Purine and pyrimidine metabolism has been proven to be closely correlated with cardiovascular disease. For instance, uric acid, the final product of purine metabolism oxidation, is known to be causally linked to adverse cardiovascular outcomes, particularly sudden cardiac death (21). In the cerebral vascular system, the relaxation of cerebral vessels induced by purine and pyrimidine nucleotides typically relies on the presence of the endothelium. Conversely, the vasoconstriction of cerebral arteries in response to ATP, UTP, and UDP is mediated by specific smooth muscle receptors, including P2X1, P2Y2, P2Y4, and P2Y6 receptors. Adenosine primarily induces vasodilation through its actions on A2A and A2B receptors present in both smooth muscle and endothelial cells. Endothelium-dependent vasodilation of cerebral arteries can be triggered by ADP via P2Y1 receptors, UTP through activation of P2Y2 and/or P2Y4 receptors, and UDP via P2Y6 receptors. Additionally, prolonged exposure to UTP has various effects on vascular endothelial cells, including chemotactic, mitogenic, and angiogenic actions (22).

In our study, purine metabolites hypoxanthine, xanthine, and xanthosine were found with significantly higher levels in MMD patients compared to HCs. Hypoxanthine and xanthine undergo oxidation catalyzed by xanthine oxidase, leading to the formation of uric acid, which represents the final product in the purine metabolism pathway. Notably, this enzymatic conversion results in the generation of reactive oxygen species (ROS) as a byproduct, which can have implications for oxidative stress and related cellular processes. In non-ischemic tissues, xanthine oxidoreductase exists primarily in the form of xanthine dehydrogenase. However, when tissues are subjected to metabolic stress, such as hypoxia or ischemia, this enzyme can undergo transformation into xanthine oxidase. Under such conditions, xanthine oxidase has the capacity to generate reactive oxygen species (ROS) including superoxide (O_2−_), hydrogen peroxide (H_2_O_2_), and hydroxyl radicals (•OH). This occurs particularly during the increased oxygenation of the tissue that takes place upon reperfusion, contributing to oxidative stress and potential tissue damage (23). MMD is characterized by complex changes in blood perfusion and intracranial ischemia during the disease process, and ROS-related endothelial mitochondrial abnormalities have been found in MMD patients (24–26). Kim et al. (27) proved that an elevated extracellular concentration of hypoxanthine has been shown to induce endothelial apoptosis by triggering the production of ROS. Additionally, it can influence the expression of proteins associated with apoptosis in human umbilical vascular endothelial cells. Currently, antioxidant agents and compounds that inhibit xanthine oxidase activity are being utilized in the treatment of various medical conditions, including hypertension, that are associated with endothelial dysfunction. These treatments aim to counteract the harmful effects of ROS and mitigate the impact on endothelial function (28–30). Although purine or hypoxanthine changes haven't been mentioned in MMD before, endothelial dysfunctions like impaired proliferation and decreased tube-forming ability have been widely reported in studies on MMD (17, 31, 32). Data from our study here provide a clue that purine metabolism may participate in the MMD pathological process via endothelial dysfunction regulation.

As for pyrimidine metabolism, pyrimidine metabolites uridine and cytidine levels were significantly higher in MMD patients than HCs. Uridine is the primary pyrimidine nucleoside extensively absorbed by the brain and functions as a neuroactive molecule, actively participating in the promotion of sleep (33, 34), anti-epilepsy (35, 36), memory improving (37, 38), and influencing neuronal plasticity (39–42). These effects depend mainly on its role in membrane formation through binding to known uridine nucleotide receptors in the plasma membrane or intracellular binding sites in the central nervous system (43). Pyrimidine metabolism, specifically the levels of uridine and cytidine, have not been extensively studied in patients with MMD and other cerebral vascular diseases (43, 44). Our study, which found that uridine and cytidine levels were significantly higher in MMD patients compared to healthy controls, represents one of the few studies on this topic. These findings suggest that pyrimidine metabolism may play a role in the development or progression of MMD. Uridine has been shown to have neuroprotective effects and improve blood flow in the brain (45). In animal studies, administration of uridine has been shown to improve blood flow in the brains of mice and reduce the risk of ischemic stroke. In addition, uridine supplementation has been shown to reduce oxidative stress, which is a contributing factor in the development of MMD.

Cytidine, as a precursor for the synthesis of the neurotransmitter acetylcholine, is involved in regulating blood flow and cerebral blood vessel tone. Studies have shown that cytidine supplementation can improve blood flow in the brain and reduce the risk of ischemic stroke. These findings suggest that the administration of cytidine may have a potential role in preventing the progression of MMD and reducing the risk of stroke.

However, the exact mechanisms by which pyrimidine metabolites, particularly uridine, and cytidine, may impact the development and progression of MMD is still not fully understood, and further studies are needed to confirm these findings and explore their potential as therapeutic options.

Except for purine metabolism and pyrimidine metabolism, the identified biomarkers also included several fatty acids, such as oleamide, hexadecanamide, and stearoyl ethanolamide (Figure 2C.) Although specific studies directly linking oleamide, hexadecanamide, and stearoyl ethanolamide to MMD are lacking, investigations into the role of fatty acids and their metabolites in vascular health provide valuable insights. Studies on omega-3 and omega-6 polyunsaturated fatty acids have revealed their significant regulatory effects on endothelial cell function and vascular contraction (46). Additionally, amide compounds such as oleamide and hexadecanamide may influence inflammation and immune system regulation by affecting cell membrane properties and signal transduction pathways (47, 48). The application of metabolomics in identifying potential biomarkers could contribute to early diagnosis and therapeutic research for MMD.

There are some limitations in this study: the study only enrolled 16 patients with MMD and eight healthy controls, which might limit the generalizability of the results to larger populations. Single-center study: The study was conducted at a single center, which may introduce biases and limit the external validity of the results. Lack of longitudinal data: The study is cross-sectional, meaning it only collected data at one point in time and does not provide information on how metabolite levels change over time. Our study only provides an association between metabolite levels and MMD but does not explain the underlying mechanisms.

## Conclusion

In conclusion, this study showed that there was a clear difference in metabolites between MMD patients and HCs. A total of 129 metabolites were identified with significant differences and were enriched in several metabolic pathways. The most affected pathways were purine metabolism and pyrimidine metabolism. The findings provide new insights into the metabolic characteristics of MMD and further research is needed to understand the mechanisms of MMD and its progression.

## Data availability statement

The original contributions presented in the study are included in the article/Supplementary material, further inquiries can be directed to the corresponding authors.

## Ethics statement

The studies involving humans were approved by Medical Ethics Committee of Zhongnan Hospital of Wuhan University. The studies were conducted in accordance with the local legislation and institutional requirements. The participants provided their written informed consent to participate in this study.

## Author contributions

JY: Funding acquisition, Investigation, Visualization, Writing – original draft, Writing – review & editing. TC: Investigation, Methodology, Validation, Writing – original draft. XL: Conceptualization, Investigation, Writing – review & editing. JC: Conceptualization, Investigation, Writing – review & editing. WW: Conceptualization, Investigation, Writing – review & editing. JZ: Conceptualization, Investigation, Writing – review & editing.
