# Supplementary figures and images for: Liquid chromatography coupled to mass spectrometry metabolomic analysis of cerebrospinal fluid revealed the metabolic characteristics of moyamoya disease
Source: Front Neurol. 2024 Feb 15;15:1298385. doi: 10.3389/fneur.2024.1298385 (PMC10902010; doi:10.3389/fneur.2024.1298385)

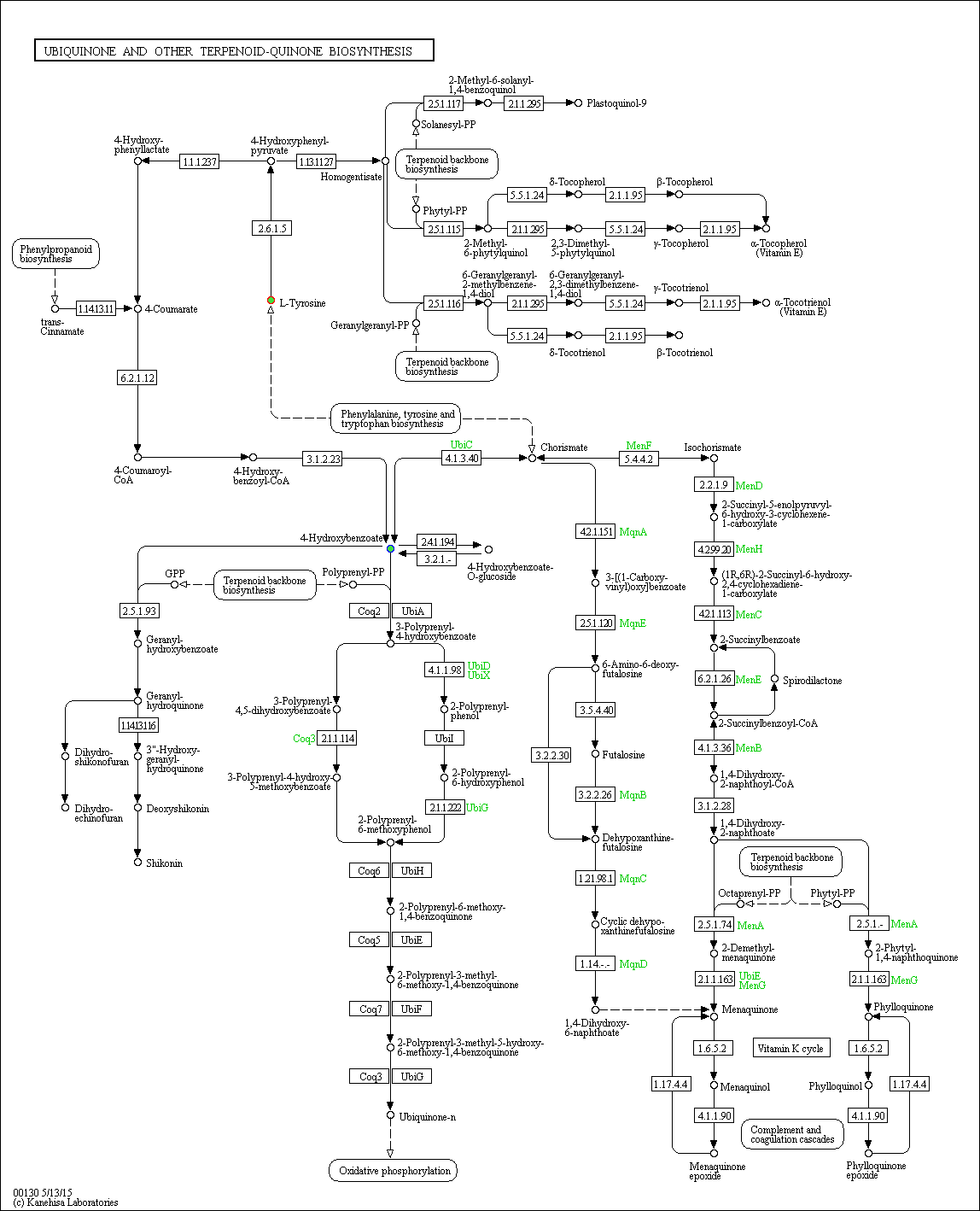

Supplement: Supplementary file 2 [file Data_Sheet_2.ZIP › Supplementary data 2:The most enriched pathway terms of MMD.vs.Ctl_all/00130.png]

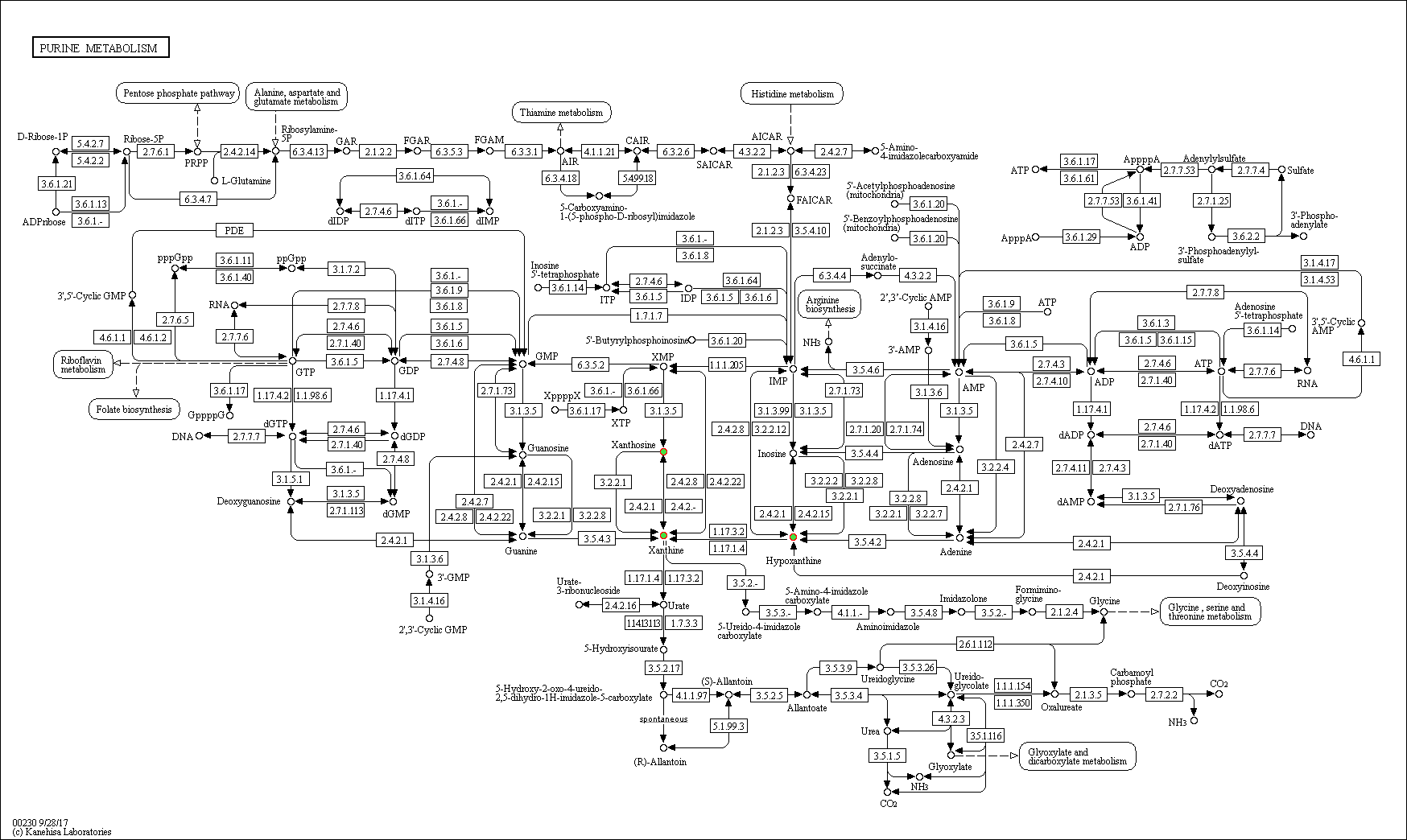

Supplement: Supplementary file 2 [file Data_Sheet_2.ZIP › Supplementary data 2:The most enriched pathway terms of MMD.vs.Ctl_all/00230.png]

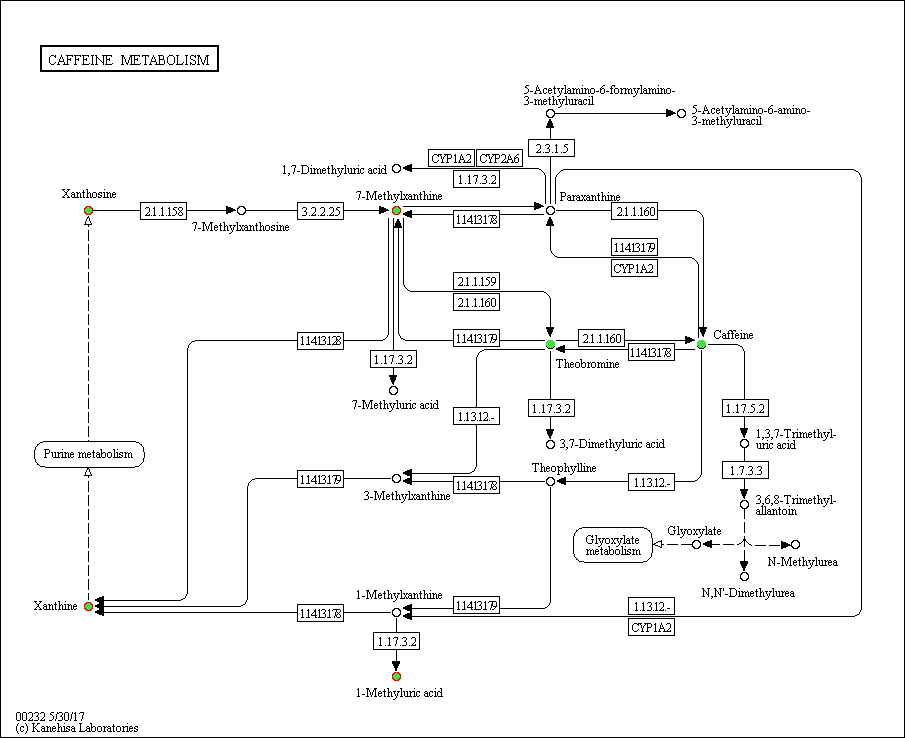

Supplement: Supplementary file 2 [file Data_Sheet_2.ZIP › Supplementary data 2:The most enriched pathway terms of MMD.vs.Ctl_all/00232.png]

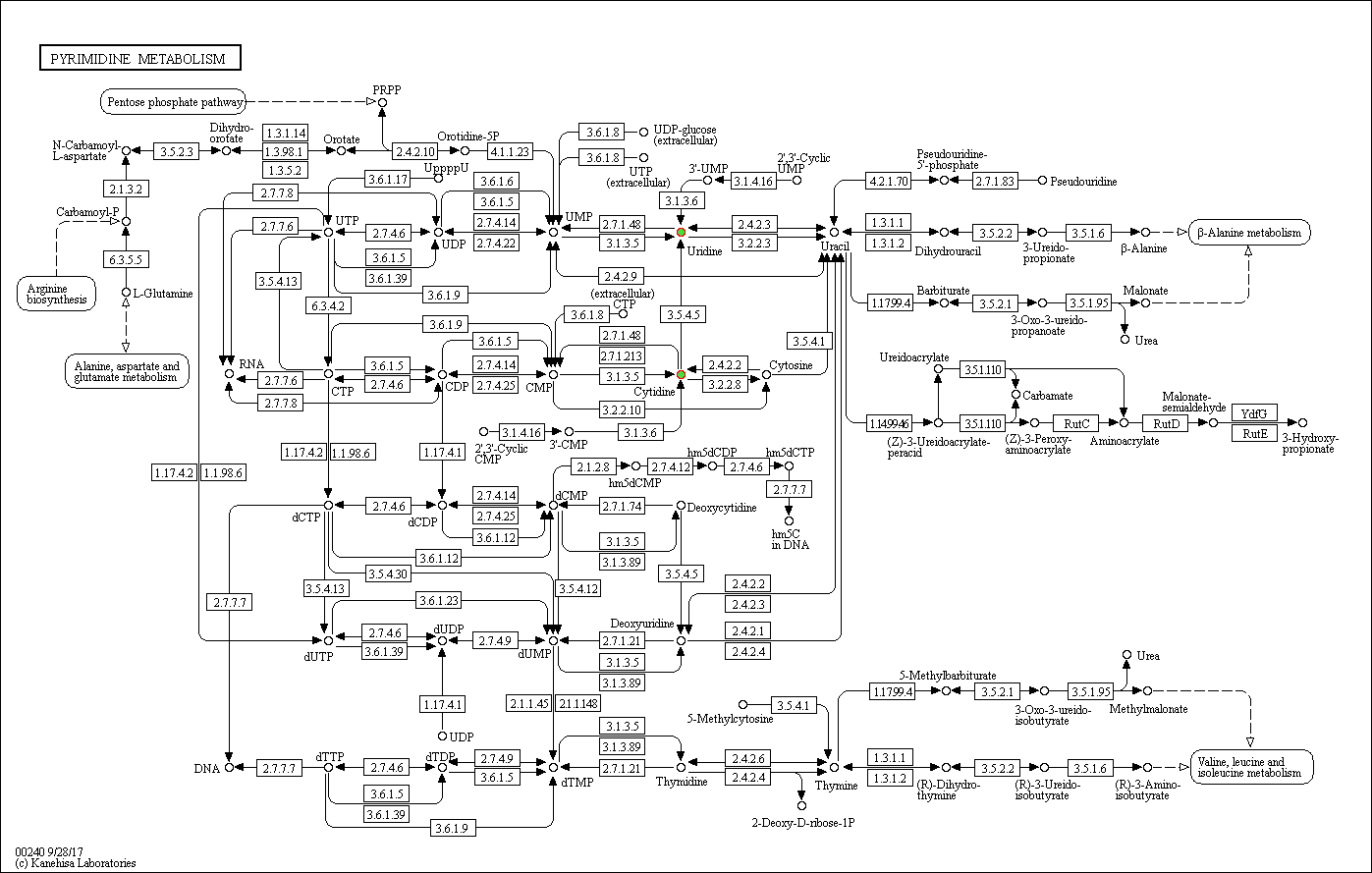

Supplement: Supplementary file 2 [file Data_Sheet_2.ZIP › Supplementary data 2:The most enriched pathway terms of MMD.vs.Ctl_all/00240.png]

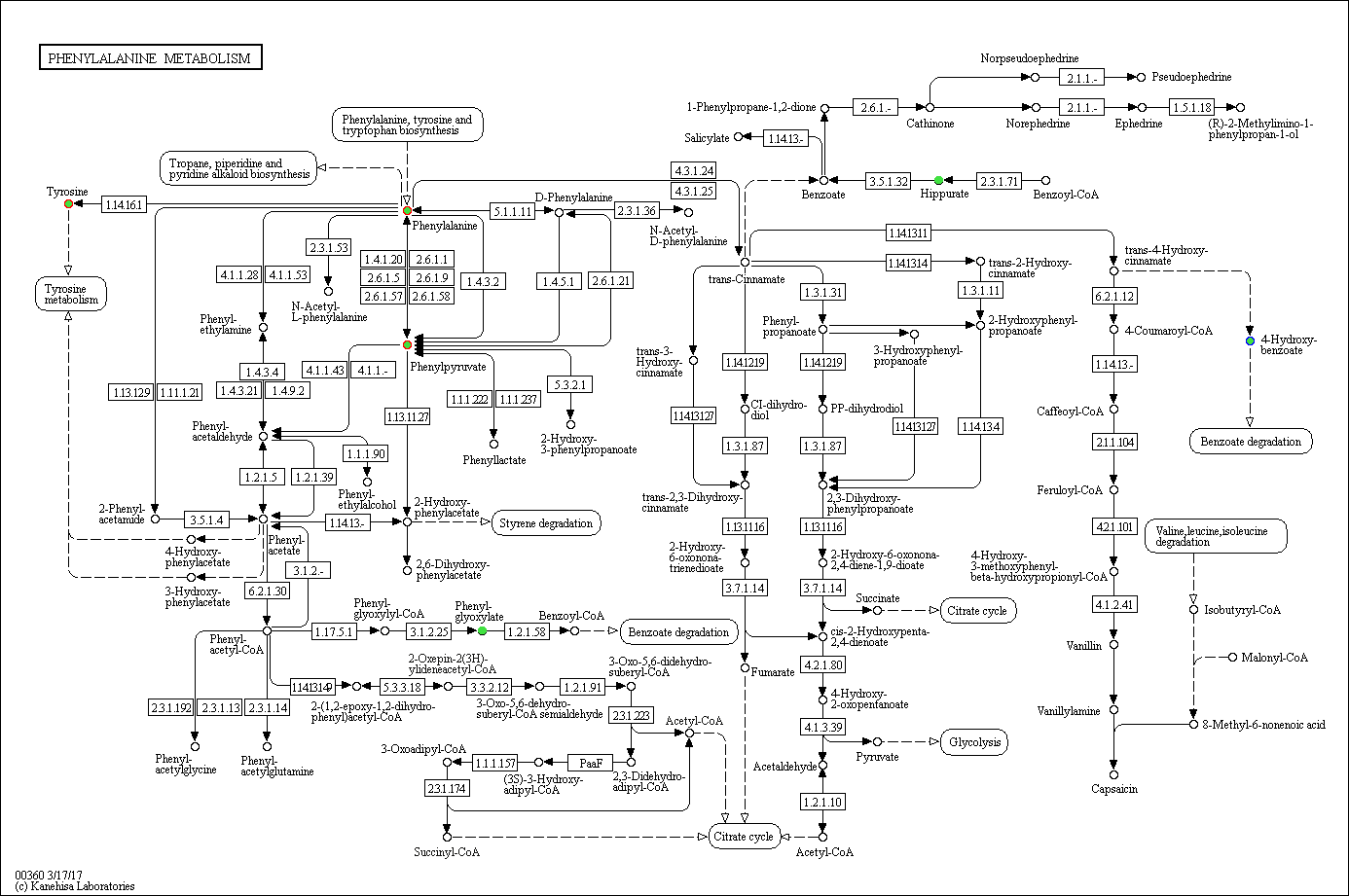

Supplement: Supplementary file 2 [file Data_Sheet_2.ZIP › Supplementary data 2:The most enriched pathway terms of MMD.vs.Ctl_all/00360.png]

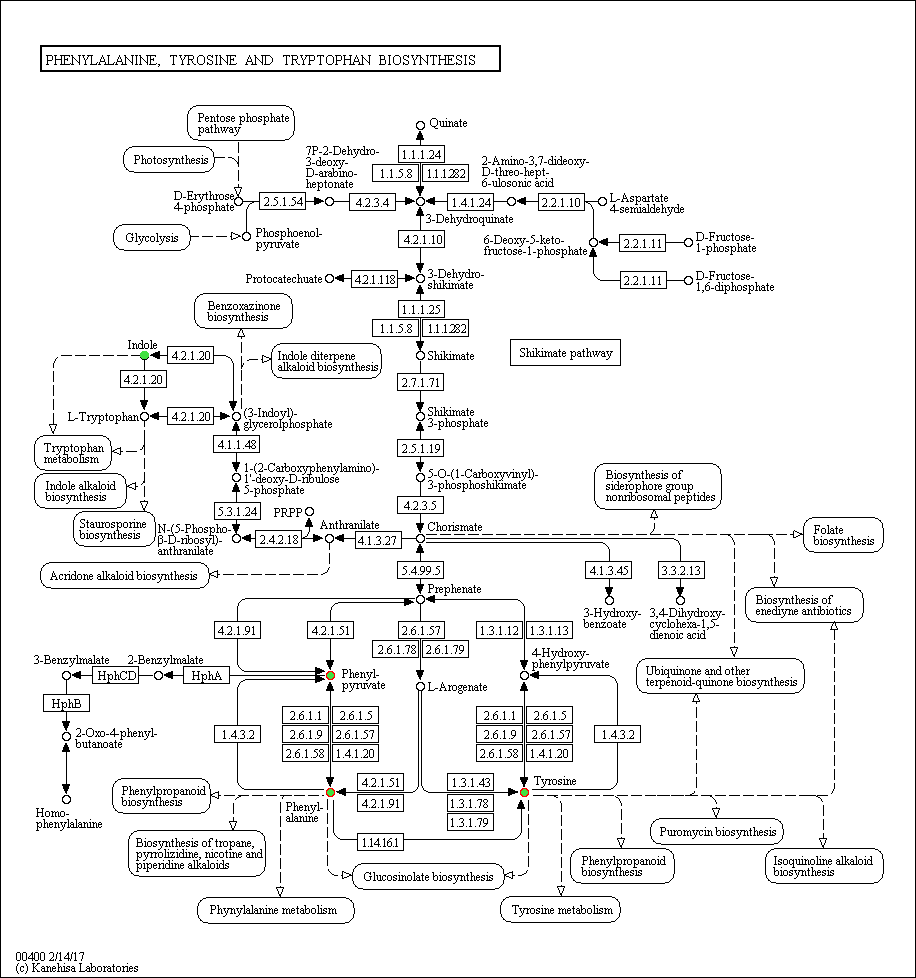

Supplement: Supplementary file 2 [file Data_Sheet_2.ZIP › Supplementary data 2:The most enriched pathway terms of MMD.vs.Ctl_all/00400.png]

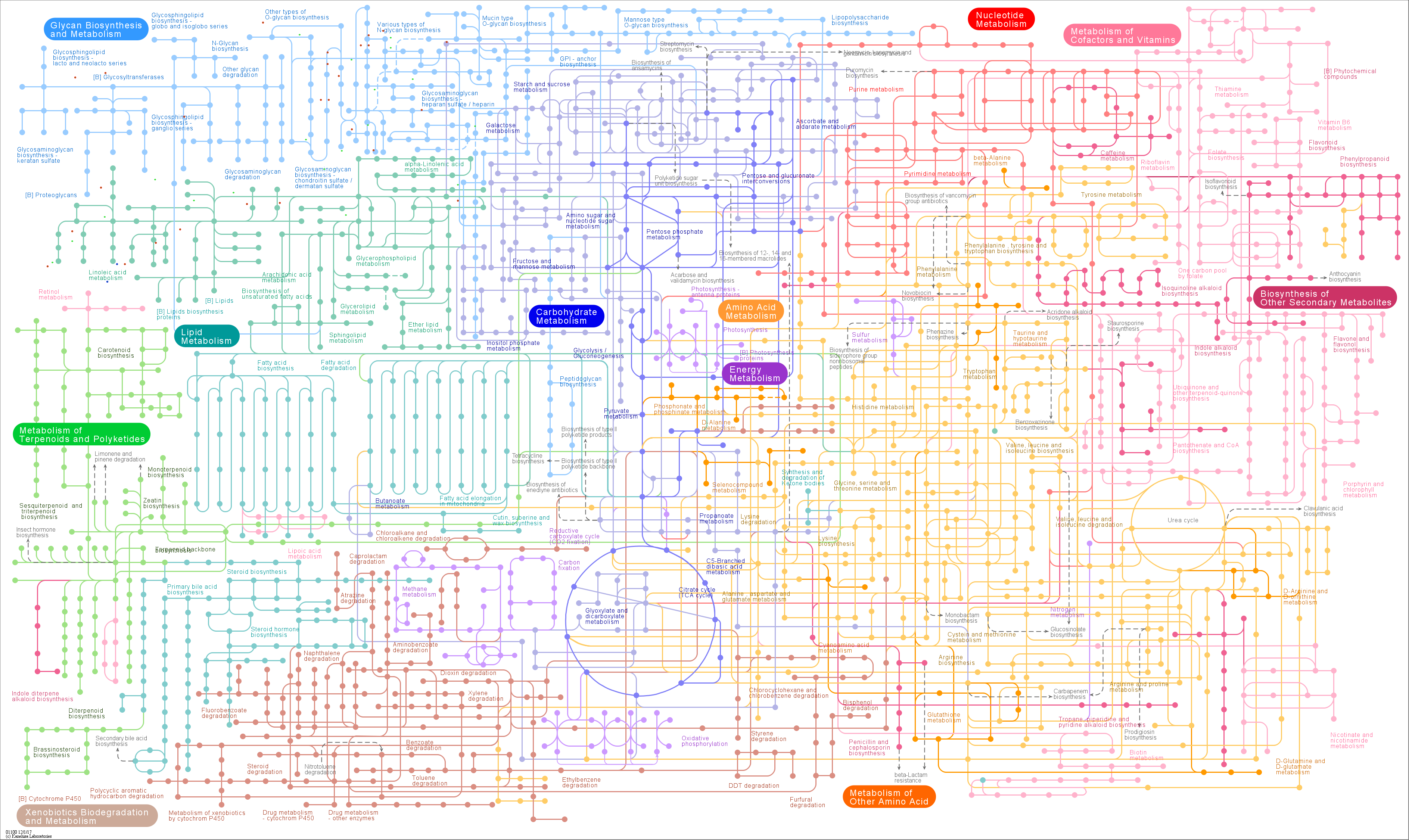

Supplement: Supplementary file 2 [file Data_Sheet_2.ZIP › Supplementary data 2:The most enriched pathway terms of MMD.vs.Ctl_all/01100.png]

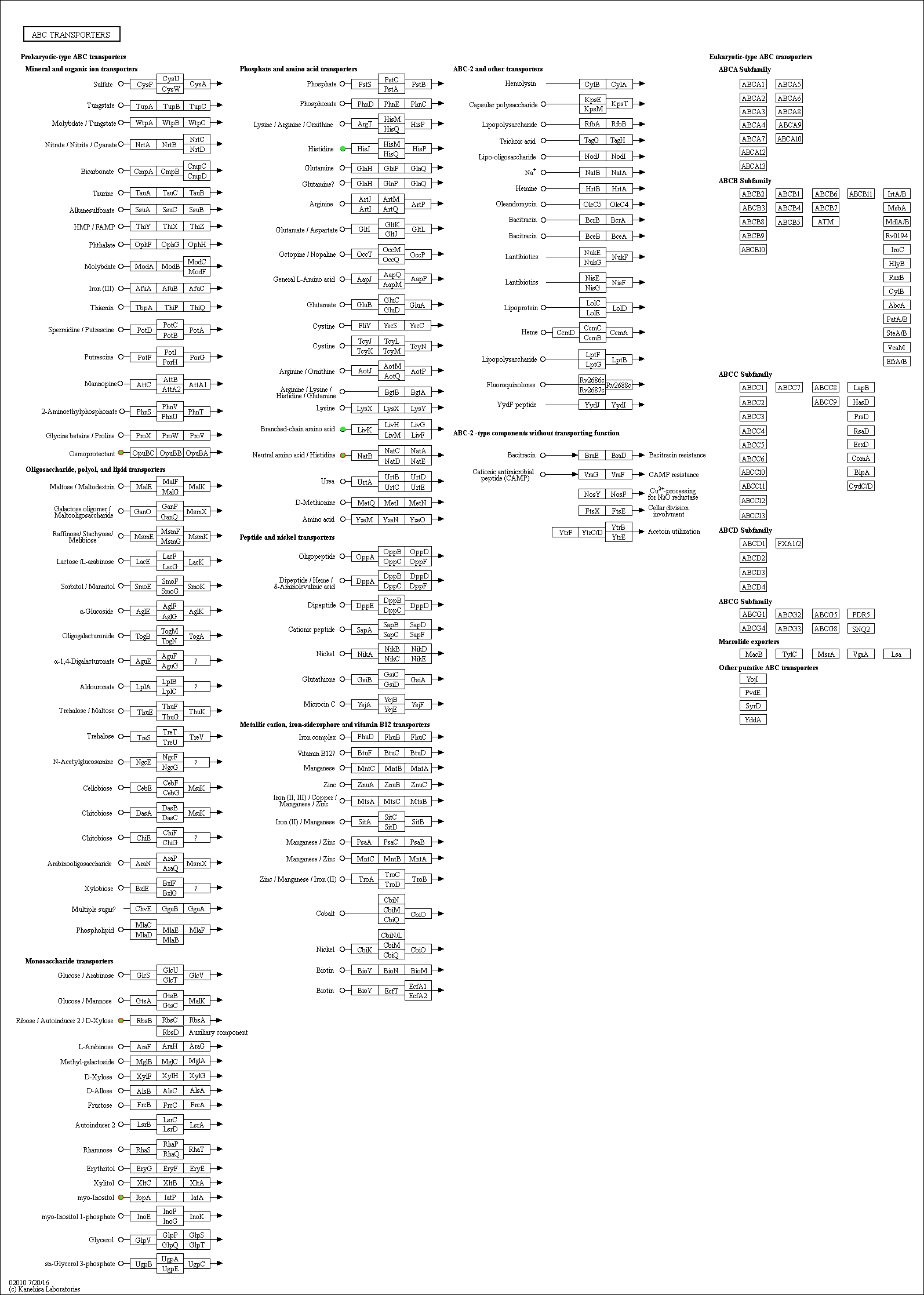

Supplement: Supplementary file 2 [file Data_Sheet_2.ZIP › Supplementary data 2:The most enriched pathway terms of MMD.vs.Ctl_all/02010.png]

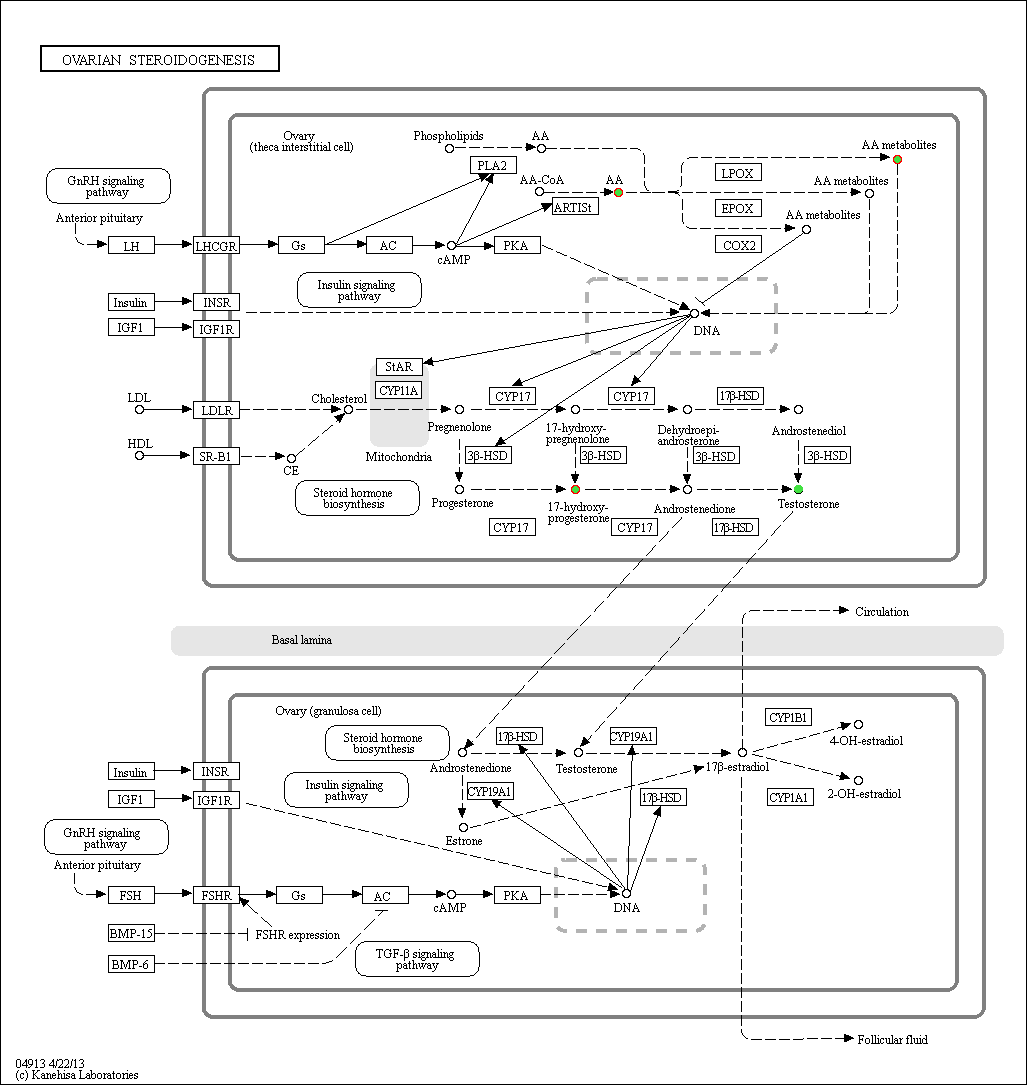

Supplement: Supplementary file 2 [file Data_Sheet_2.ZIP › Supplementary data 2:The most enriched pathway terms of MMD.vs.Ctl_all/04913.png]

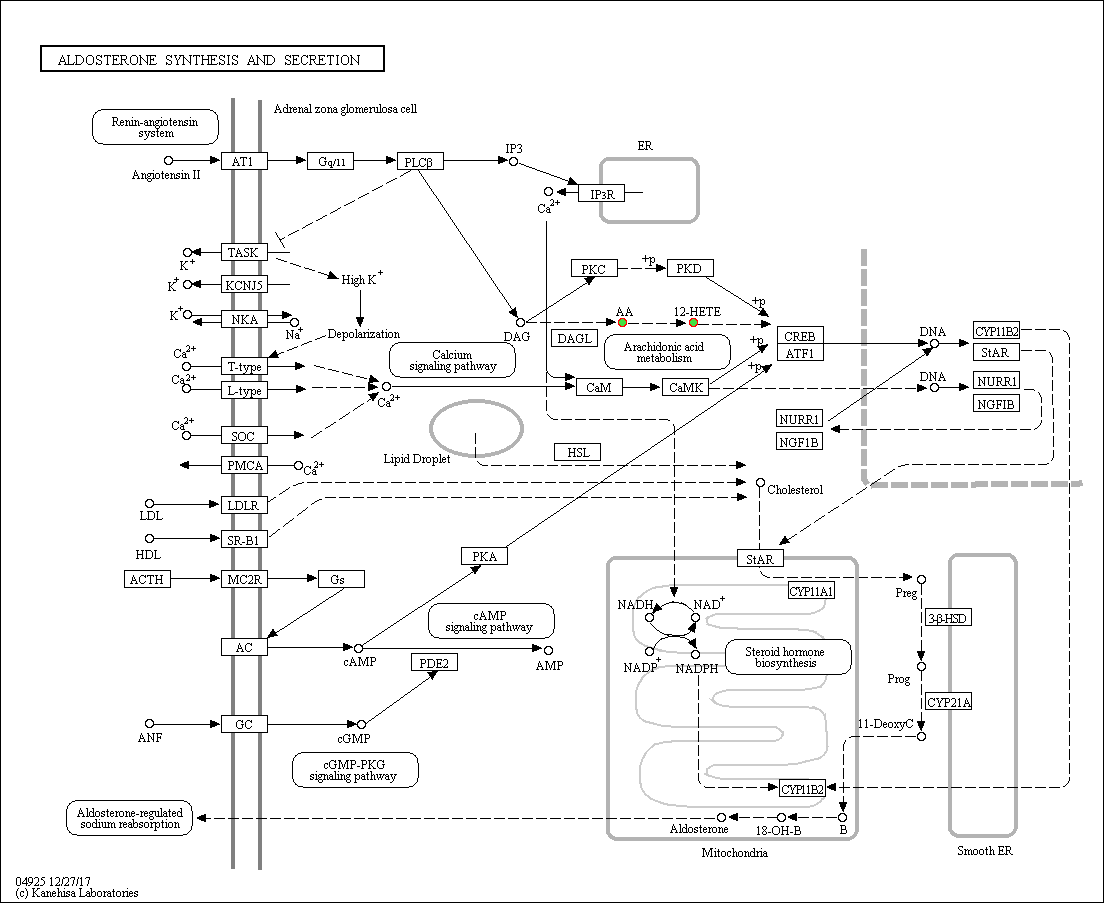

Supplement: Supplementary file 2 [file Data_Sheet_2.ZIP › Supplementary data 2:The most enriched pathway terms of MMD.vs.Ctl_all/04925.png]
